# Antibody responses against the vaccine antigens *Ov*-103 and *Ov*-RAL-2 are associated with protective immunity to *Onchocerca volvulus* infection in both mice and humans

**DOI:** 10.1371/journal.pntd.0007730

**Published:** 2019-09-16

**Authors:** Parakkal Jovvian George, Jessica A. Hess, Sonia Jain, John B. Patton, Tingting Zhan, Nancy Tricoche, Bin Zhan, Maria Elena Bottazzi, Peter J. Hotez, David Abraham, Sara Lustigman

**Affiliations:** 1 Laboratory of Molecular Parasitology, Lindsley F Kimball Research Institute, New York Blood Center, New York, New York, United States of America; 2 Department of Microbiology and Immunology, Sidney Kimmel Medical College, Thomas Jefferson University, Philadelphia, Pennsylvania, United States of America; 3 Division of Biostatistics, Department of Pharmacology and Experimental Therapeutics, Thomas Jefferson University, Philadelphia, Pennsylvania, United States of America; 4 Texas Children’s Hospital Center for Vaccine Development, Departments of Pediatric Tropical Medicine and Molecular Virology and Microbiology, National School of Tropical Medicine, Baylor College of Medicine, Houston, Texas, United States of America; University of Zurich, SWITZERLAND

## Abstract

**Background:**

The current strategy for the elimination of onchocerciasis is based on annual or bi-annual mass drug administration with ivermectin. However, due to several limiting factors there is a growing concern that elimination of onchocerciasis cannot be achieved solely through the current strategy. Additional tools are critically needed including a prophylactic vaccine. Presently *Ov*-103 and *Ov*-RAL-2 are the most promising vaccine candidates against an *Onchocerca volvulus* infection.

**Methodology/Principal findings:**

Protection induced by immunization of mice with the alum-adjuvanted *Ov*-103 or *Ov*-RAL-2 vaccines appeared to be antibody dependent since AID^-/-^ mice that could not mount antigen-specific IgG antibody responses were not protected from an *Onchocerca volvulus* challenge. To determine a possible association between antigen-specific antibody responses and anti-larvae protective immunity in humans, we analyzed the presence of anti-*Ov*-103 and anti-*Ov*-RAL-2 cytophilic antibody responses (IgG1 and IgG3) in individuals classified as putatively immune, and in infected individuals who developed concomitant immunity with age. It was determined that 86% of putatively immune individuals and 95% individuals with concomitant immunity had elevated IgG1 and IgG3 responses to *Ov*-103 and *Ov*-RAL-2. Based on the elevated chemokine levels associated with protection in the *Ov*-103 or *Ov*-RAL-2 immunized mice, the profile of these chemokines was also analyzed in putatively immune and infected individuals; both groups contained significantly higher levels of KC, IP-10, MCP-1 and MIP-1β in comparison to normal human sera. Moreover, human monospecific anti-*Ov*-103 antibodies but not anti-*Ov*-RAL-2 significantly inhibited the molting of third-stage larvae (L3) *in vitro* by 46% in the presence of naïve human neutrophils, while both anti-*Ov*-103 and anti-*Ov*-RAL-2 antibodies significantly inhibited the molting by 70–80% when cultured in the presence of naive human monocytes. Interestingly, inhibition of molting by *Ov*-103 antibodies and monocytes was only in part dependent on contact with the cells, while inhibition of molting with *Ov*-RAL-2 antibodies was completely dependent on contact with the monocytes. In comparison, significant levels of parasite killing in *Ov*-103 and *Ov*-RAL-2 vaccinated mice only occurred when cells enter the parasite microenvironment. Taken together, antibodies to *Ov*-103 and *Ov*-RAL-2 and cells are required for protection in mice as well as for the development of immunity in humans.

**Conclusions/Significance:**

Alum-adjuvanted *Ov*-103 and *Ov*-RAL-2 vaccines have the potential of reducing infection and thus morbidity associated with onchocerciasis in humans. The development of cytophilic antibodies, that function in antibody-dependent cellular cytotoxicity, is essential for a successful prophylactic vaccine against this infection.

## Author summary

*Onchocerca volvulus* is the causative agent of river blindness that infects approximately 17 million people, mostly in Africa. The current strategy for elimination of *O*. *volvulus* focuses on controlling transmission through ivermectin-based mass drug administration programs. Due to potential ivermectin resistance, the lack of macrofilaricidal activity by ivermectin, and the prolonged time (>20 years) needed for successful interruption of transmission in endemic areas, additional tools are critically needed including a vaccine against onchocerciasis. *Ov*-103 and *Ov*-RAL-2 are presently the most promising vaccine candidates for a prophylactic vaccine. The mechanism of protective immunity induced in mice by the alum-adjuvanted *Ov*-103 or *Ov*-RAL-2 vaccines appear to be multifactorial with essential roles for antibodies, chemokines and the specific effector cells they recruit. In this study, we show for the first time that, anti-*Ov*-103 and anti-*Ov*-RAL-2 antibodies, chemokines and innate cells also appear to be associated with protective immunity against *O*. *volvulus* infection in humans, similar to the vaccine studies observed in the *O*. *volvulus* mouse model.

## Introduction

*Onchocerca volvulus*, a filarial nematode, is the etiologic agent of river blindness that infects approximately 17 million people in Africa with more than 10 million people living with skin disease and 1 million with visual impairment [1]. The current strategy for elimination of *O*. *volvulus* focuses on controlling transmission through ivermectin-based mass drug administration (MDA) programs. Due to factors such as the possible development of drug resistance, the need for lengthy (>20 years) annual drug administration, the inability to implement large-scale treatment programs in areas that are co-endemic for loiasis, it remains unlikely that onchocerciasis can be eliminated entirely through MDA with only ivermectin [2]. This realization has stimulated the search for companion intervention tools, including vaccines, to support the efforts to eliminate onchocerciasis [3–5].

A multinational consortium and initiative known as TOVA (The Onchocerciasis Vaccine for Africa) is working to develop a prophylactic recombinant subunit vaccine to supplement the MDA programs [3–5]. Currently, the lead candidate vaccine is comprised of two recombinant *O*. *volvulus* antigens, *Ov*-103 and *Ov*-RAL-2 [4]. Both antigens were shown to elicit protection in a mouse model of *O*. *volvulus* infection with third-stage larvae (L3) [6, 7]. Similarly, the *Brugia malayi* orthologous antigens were protective in a *B*. *malayi*-gerbil model, where the full life cycle of the parasite is known to develop [8].

The precise mechanisms by which protected mice and humans exposed to infection can kill larval stages of *O*. *volvulus* have not yet been fully defined. In general, it is thought that the killing of helminth parasites, which are macropathogens, is mediated by granulocytes, macrophages and antibodies using antibody-dependent cellular cytotoxicity (ADCC). The Fc-receptor-bearing effector cells can recognize and kill antibody-coated parasite worms by discharging their lysosomal or granular content (reviewed in [9–11]). In mice, immunization with irradiated L3 of *O*. *volvulus* induced a protective mechanism that is dependent on IgE and eosinophils [12]. Protection in mice induced by immunization with alum-adjuvanted *Ov*-103, *Ov*-RAL-2 or *Ov*-103 co-administered with *Ov*-RAL-2 vaccines appeared to be associated with a multifactorial complex network of immune factors including specific antibodies, Th2 cytokines, chemokines, and possibly specific effector cells recruited by the chemokines such as neutrophils, monocyte/macrophages and/or eosinophils [7]. Naïve human neutrophils in the presence of polyclonal antibodies from sera samples of *O*. *volvulus* infected and putatively immune individuals have shown to be effective at killing L3 and microfilariae of *O*. *volvulus in vitro* [13, 14]. In the gerbil-*Brugia malayi* infection animal model, protection induced by immunization with *Bm*-RAL2 and *Bm*-103 vaccine antigens was in part associated with the presence of antigen-specific functional antibodies that could kill *B*. *malayi* L3 *in vitro* in the presence of peritoneal exudate cells [8]. Notably, in both *B*. *malayi* and *O*. *volvulus*, the native proteins corresponding to *Bm*-RAL2, *Ov*-RAL2, *Bm-103* and *Ov*-103 are expressed in the hypodermis of L3 and on the surface of microfilariae (mf) [8, 15], and human monospecific antibodies against *Ov*-103 can kill mf *in vitro* in the presence of neutrophils [15]. Previous studies have also shown that protective immunity in humans against L3 is associated with mixed Th1 and Th2 cytokine responses, elevated IgG1, IgG3 and IgE cytophilic antibody responses, and possibly ADCC [16–18].

The objective of the present study was to determine whether the anti-*Ov*-103 and anti-*Ov*-RAL-2 antibody responses elicited in the vaccinated mice are essential for protective immunity. In addition, we tested whether the *Ov*-103 and O*v*-RAL-2 antigen-specific cytophilic antibodies are also associated with protective immunity to infections with the L3 that develops in humans exposed to *O*. *volvulus* infections, i.e. in putatively immune individuals (individuals exposed to high transmission rates of infection but had no signs or history of clinical manifestations of onchocerciasis and were negative for the presence of the *O*. *volvulus* specific 150-mer DNA repeat in skin snips over five years of surveillance) [18], and in infected individuals who develop concomitant immunity with age (protection that limits newly acquired infections while adult worms and mf are not affected [17]. We also tested *in vitro* whether these antibodies are functional in ADCC in the presence of naïve human monocytes and neutrophils.

## Materials and methods

### Ethics statement

All the animals in this study were handled according to the National Institutes of Health (USA) guidelines. The animal experimentation was performed with prior approval from the Institutional Animal Care and Use Committee of Thomas Jefferson University under the protocol number 00136.

Male C57BL/6J and B6.129P2-*Aicda*^*tm1(cre)Mnz*^/J(AID^-/-^) mice at 6–8 weeks of age were purchased from The Jackson Laboratory (Bar Harbor, Maine). Mice were kept in the Thomas Jefferson University Laboratory Animal Sciences Facility. All mice were housed in micro-isolator boxes in a room that was pathogen free and under temperature, humidity and light cycle-controlled conditions. Mice were fed autoclavable rodent chow and given water ad libitum.

The protocols used in all the human population studies were approved by the Institutional Review Board (IRB) of the New York Blood Center’s IRB and by the National Institutes of Health (USA) accredited Institutional Review Board of the Medical Research Council Kumba, Cameroon (Kumba studies). Informed written consent was obtained from all adult subjects, and for children consent was obtained through both verbal assent and written consent from each subject’s legal guardian.

The serum samples used for the present studies were from a repository of frozen serum samples collected during a larger clinical study performed in 1995–2000 in Kumba, an area of hyperendemicity for onchocerciasis in southwest Cameroon. The characteristics of the populations were already described in previous publications [17, 18]. Briefly, the consenting participants enrolled in the study were born or have resided for more than 10 years in their respective villages (Marumba I, Marumba II, Boa Bakundu, Bombanda, and Bombele) in Cameroon. The standard skin snip test for detection of mf was performed on each subject and clinical symptoms of onchocerciasis were recorded. The average number of mf in four skin snips taken from each individual were used in estimating the individual skin mf densities. Individuals who were diagnosed clinically as infected (presence of nodules and/or other clinical manifestations of onchocerciasis) but were negative for skin mf were confirmed to be infected (INF) by establishing the presence of *O*. *volvulus* 150-mer DNA tandem repeat in the skin snips using a polymerase chain reaction (PCR) followed by Southern blotting using a specific internal *O*. *volvulus* probe [17, 18]. Individuals were classified as putatively immune (PI) if they had no skin mf and signs of history of onchocerciasis, as well as parasitologically (mf negative and PCR negative) and clinically negative for *O*. *volvulus* infection during a five-year follow-up survey [17, 18]. Notably, 75% of the PI individuals had IgG4 antibodies to *Ov*-16 antigen (the antigen was provided by Dr. Thomas Nutman, NIH-NIAID), which confirmed that most of the PI individuals were exposed to *O*. *volvulus* infection. In comparison, 90% of the INF individuals had IgG4 antibodies to *Ov*-16; *Ov*-16 ELISA is highly specific (>99%) but with only 60–80% sensitivity of onchocerciasis. *Ov*-16 IgG4 responses are used to measure exposure to infections with L3 of *O*. *volvulus*; the antibody response can be detected 3 months and at more than 1 year before infection could otherwise be detected [19, 20]. None of the subjects had received ivermectin treatment prior to the collection of blood. However, the consenting participants were offered and provided with ivermectin treatment by the end of their participation in the study.

The procedures used to produce *O*. *volvulus* L3 were approved by an NIH accredited Institutional Review Board of the Medical Research Council Kumba, Cameroon (Protocol 001), and by the Le Comité National d’Ethique de la Recherche pour la Santé Humaine, Yaoundé, Cameroon (Protocol 677). L3 were collected from black flies (*Simulium damnosum*) that were fed on consenting infected donors. The consenting donors were offered and provided with ivermectin treatment by the end of their participation. After seven days the infected flies were dissected and the developed L3 were collected, cleaned and cryopreserved. The cryopreserved L3 were shipped to the New York Blood Center in liquid nitrogen and upon arrival in New York were stored in liquid nitrogen. All protocols using the L3 cryopreserved samples in this study were approved by the New York Blood Center’s IRB (Protocol 321 and Protocol 603–09). All L3 samples were anonymized. Informed written consent was also obtained from donors who donated blood and reside in the U.S. The protocol was approved by the New York Blood Center’s IRB (Protocol 420). The sera from these donors were treated as normal healthy controls (NH).

### Production of antigens

Based on previous studies, *Ov*-103 was expressed in PichiaPink yeast and *Ov*-RAL-2 was expressed in *Escherichia coli*. The recombinant antigens were prepared and analyzed as previously described [6]. Residual endotoxin was removed from the *E*. *coli* expressed *Ov*-RAL-2 using a Q anion exchange column. The level of endotoxin in the final products of both recombinant proteins was less than 20 EU/mg (13.2–19.3 EU/mg).

### Immunizations

Mice were immunized with 25 μg of the recombinant vaccine antigen formulated with 200 μg Rehydragel LV (alum, General Chemical, Parsippany, NJ) followed by two booster injections 14 and 28 days later. For the *Ov*-103 or *Ov*-RAL-2 alum-adjuvanted vaccines, 25 μg of each antigen in TBS was mixed 1:1 v/v with 1:5 in TBS diluted alum in a total volume of 100 μL. Mice were immunized by intramuscular injection into each caudal thigh with 50 μL of the formulated vaccine antigens.

### Challenge of mice with *O*. *volvulus* L3 within diffusion chambers

Challenge infections occurred 14 days after the final booster with 25 L3 within a diffusion chamber. Cryopreserved L3 were defrosted slowly in a two-step process, first 15 minutes on dry ice followed by a 37°C water bath. Once thawed the L3 were washed 5 times in a 1:1 mixture of NCTC-135 and Iscove’s modified Dulbecco’s medium (Sigma, St. Louis, MO) containing 100 U penicillin,100 μg streptomycin (Corning, Tewksbury, MA), 100 μg gentamicin and 30 μg of chloramphenicol per ml (Millipore Sigma, St. Louis, MO). Diffusion chambers were constructed using 14 mm Lucite rings covered with either 5.0 or 0.1 μm pore-size Durapore membranes (EMD Millipore, Billerica, MA) and fused together using an adhesive containing a 1:1 mixture of 1,2-dichloroethane (Fisher Scientific, Pittsburg, PA) and acryloid resin (Rohm and Haas, Philadelphia, PA). The constructed diffusion chambers were sterilized using 100% ethylene oxide followed by 12 hr aeration.

The diffusion chambers were implanted in a subcutaneous pocket on the rear flank of the mice. Recovery of the diffusion chambers was performed 21 days later, and larval survival was determined based on mobility and morphology of the remaining larvae. Protective immunity was calculated based on survival of larvae within the diffusion chambers: Percent reduction = [(Average worm survival in control mice—Average worm survival in immunized mice) ÷ Average worm survival in control mice] × 100.

The surviving larvae were fixed in hot 95% EtOH (Sigma, St. Louis, MO) and 5% Glycerol (Fisher Scientific) and measured using CellSens Dimension software (Olympus, Center Valley, PA). Host cells within the diffusion chamber were collected and analyzed by centrifugation onto slides using a Cytospin 3 (Shandon Inc, Pittsburgh, PA) and then stained for differential cell counts using Hemastain 3 (Fisher Scientific, Pittsburg, PA).

### Antibody responses to *Ov*-103 and *Ov*-RAL-2 in immunized mice

Serum was collected at the end of the experiment for antigen specific IgM and IgG1 analysis. Maxisorp 96-well plates (Nunc Nalgene International, Rochester, NY) were coated with 2 μg/ml of the immunizing recombinant antigen in 50 mM Tris-HCl coating buffer pH 8.8 overnight 4°C. Plates were washed with deionized water between each step. Plates were blocked with borate buffer solution (BBS) (0.17 M boric acid, 0.12 M NaCl, 0.5% tween 20, 0.025% bovine serum albumin, 1 MM EDTA, pH 8.2) at room temperature for 30 min. Individual sera were diluted to an appropriate starting concentration with BBS, serially diluted and then added to the wells. Plates were sealed and incubated at 4°C overnight. Biotinylated anti-mouse IgM and anti-mouse IgG1 (eBioscience, San Diego, CA) was diluted 1:250 in BBS and incubated for 1 hr at room temperature, followed by incubation with ExtrAvidin Px (Sigma) 1:1000 in BBS for 30 min at room temperature. One component ABTS peroxidase substrate (KPL, Gaithersburg, MD) was added and optical densities were read after 30 min at 405 nm in a Bio-Rad iMark Microplate reader (Bio-Rad, Hercules, CA). ELISA data is presented as endpoint titers which were calculated as the serum dilution from experimental animals that had an optical density reading three times higher than the optical density recorded for control serum.

### Cytokine responses in the immunized mice

One week after diffusion chamber recovery, spleens were aseptically removed from all experimental mice and homogenized into a single cell suspension. After RBC lysis, 2 x 10^6^ cells were cultured in 96 well plates with either 10 μg of *Ov*-103, *Ov*-RAL-2, media or anti-CD3 mAb (BD Biosciences, San Jose, CA). For a positive control, wells were pre-coated with anti-CD3 (0.5 μg/ml) overnight at 4°C, this is the internal control to ensure the assay has worked appropriately and all T cells are generically stimulated. Each well in the assay also received 0.5 μL of anti-IL-4r Ab (BD Biosciences). The use of 0.5 μL of anti-IL-4r antibody in each well is used so that the soluble IL-4 produced by cells does not immediately bind to cells and become unmeasurable in the assays. As IL-4, IL-5, and other TH-2 cytokines are important in the model, this is necessary to use to get quantifiable data [21–23]. Cells were incubated at 37°C for 3 days, when supernatants were collected and frozen at -20°C until use.

Supernatants from the stimulated spleen cells were analyzed using Milliplex Map Kit magnetic bead panels as per the manufacturer’s directions (Millipore Sigma) and MAG-PIX Luminex machine (Austin, TX). The results were analyzed and calculated using Milliplex Analyst software (EMDMillipore). A 4-analyte cytokine panel was used to analyze the spleen cell supernatants.

### Antibody responses to *Ov*-103 and *Ov*-RAL-2 in putatively immune and in infected individuals

In this study we used 167 frozen sera samples collected from infected individuals (112 males and 55 females, ranging in age from 3 to 75 years). The infected population (INF) included individuals who had clinical manifestations of onchocerciasis and positive for microfilariae in their skin snip biopsies (mf+; N = 109, range of mf: 3–388 per skin snip), or those negative for skin microfilariae microscopically but positive for the *O*. *volvulus* 150-mer DNA repeat (mf-PCR+; N = 58) [18]. In addition, we analyzed serum samples of 21 putatively immune (PI) subjects and age matched infected individuals (n = 21) ranging in age from 4 to 59 years. These individuals were also assessed during enrollment for the presence of other filarial infections endemic to this region (South West region of Cameroon) such as *Loa loa* and *Mansonella perstans*. Both PI and INF individuals were negative for *L*. *loa*, while ~40% of the PI and ~20% of the INF were positive for *M*. *perstans* microfilariae.

In our previous studies using serum samples from the same populations we have shown that protective immunity in both human populations against L3 is associated with elevated IgG1, IgG3 and/or IgE cytophilic antibody responses against crude extracts and *O*. *volvulus* larval vaccine antigens [16–18, 24, 25]. Accordingly, sera obtained from INF and PI individuals were analyzed for IgG1 and IgG3 isotype antibody responses using recombinant *Ov*-103 and *Ov*-RAL-2 antigens and our established ELISA protocol [17, 26]. *Ov*-103 (1 μg/ml) and *Ov*-RAL-2 (1 μg/ml) were used to coat the wells of ELISA plates, and sera samples at 1:100 dilution were added to the bound antigens (due to the very limited volumes of sera and multiple assays that had to be performed, we were limited to single dilutions and did not perform serial dilutions to calculate endpoint titers). Bound antibodies were detected using a 1:1,000 dilution of monoclonal antibodies against IgG1 or IgG3 human subclass antibodies (Hybridoma Reagent Laboratory, Kingsville, Md.). This step was followed by incubation with a 1:1,250 dilution of horseradish peroxidase-conjugated rabbit anti-mouse immunoglobulins (Kierkegaard & Perry Laboratories, Inc., Gaithersburg, Md.). Tetramethylbenzidine (Sigma) was used as the substrate for all ELISAs, and the optical density (OD) was read at 450 nm.

### Chemokine levels in the plasma of putatively protected and infected human population

Concentrations of 8 chemokines (Eotaxin, GM-CSF, KC, IP-10, MCP-1, MIP-1α, MIP-1β and RANTES) were simultaneously quantified using a MILLIPLEX MAP human cytokine/chemokine magnetic bead panel (Millipore Sigma, Massachusetts) in plasma from the PI (N = 18) individuals as well as in INF who we classified as those with concomitant immunity (N = 17). The INF with concomitant immunity are a subset of the 109 mf+ INF individuals having high levels of mf (mean: 144.5), above the age of 11 (median: 19), and having high IgG3 antibodies responses to both antigens [mean: 0.8 (*Ov*-103) and 0.9 (*Ov*-RAL-2)].

Plates were prepared as per the manufacturer’s directions. Chemokine concentrations were measured using MagPix Luminex100 reader. The Mean Fluorescent Intensities (MFI) were analyzed using MILLIPLEXTM Analyst Software (EMD Millipore). The chemokine levels are expressed as pg/ml. Individuals with a positive chemokine response are defined as individuals having chemokine levels of more than the mean + three times the standard deviation (mean+3*SD) levels present in two pools of control normal-human sera.

### *In vitro* inhibition of L3 molting using human monospecific antibodies and naive human neutrophils or monocytes

Monospecific human antibodies to recombinant *Ov*-103 and *Ov*-RAL-2 were purified as described by Lustigman *et al*. [15, 24, 27]. Briefly, 2 mg of recombinant *Ov*-103 or *Ov*-RAL-2 were coupled to CNBr-Sepharose 4B using the protocol provided by the manufacturer (Pharmacia). Antibodies from 25 ml of pooled plasma from four infected individuals, who had high titers of anti-*Ov*-103 and *Ov*-RAL-2 antibodies, were affinity purified on the immobilized polypeptides. The mono-specificity of the eluted antibodies was confirmed by Western blot analysis. Vaccine antigen-specific negative antibodies were purified from the same pooled donor plasma using an affinity column containing an *O*. *volvulus* recombinant protein (r*Ov*-ASP-1) a putative vaccine antigen that was not able to induce protection in mice consistently [6]. The IgG endpoint titers of the anti-*Ov*-103 and *Ov*-RAL-2 purified monospecific antibodies were 1:500,000 and 1:1,000,000, respectively, as determined by ELISA, while the anti-*Ov*-ASP-1 negative control antibodies had an anti-*Ov*-103 and *Ov*-RAL-2 cross reacting antibody titers of 1∶3,200. The purified antibodies were passed through a 0.22 μM filter for sterilization.

Human neutrophils purified from naïve PBMCs were used in the inhibition of L3 molting assays as described by Johnson EH *et al*. [13]. Monocytes were purified from normal human PBMCs by positive selection using anti-CD14–conjugated magnetic microbeads (Miltenyi Biotec). L3 were washed in media that contains 1:1 NCTC-109 and IMDM supplemented with Glutamax (1x) and 2x Antibiotic-Antimycotic (Life Technologies). The larvae were diluted to 15 worms per 50 μL in serum-free media and distributed to designated wells of a 96-well plate per treatment group. 2×10^5^ normal human neutrophils or 2×10^5^ normal human monocytes were added to each well in 50 μL of complete medium with final concentration of 20% non-heat inactivated fetal calf serum (Sigma). The anti-*Ov*-103 or anti-*Ov*-RAL-2 monospecific antibodies (25 μL of endpoint titer of 1:500,000 per well), or equivalent volume of anti-O*v*-ASP-1 monospecific antibodies (non-specific negative control antibodies) were then added to each of the designated wells. Complete media without antibodies was also included as a control. Total volume per well was 200 μL. The 96-well plates were incubated at 37°C in a 5% CO_2_ incubator. Pooled sera from three normal healthy individuals (NH) and pooled sera from three individuals infected with *O*. *volvulus* who had high IgG antibody titers to both *Ov*-103 and *Ov*-RAL-2 were also used as negative and positive controls, respectively. Cultures were observed under an inverted microscope for L3 molting, the presence of the fourth-stage larvae (L4) and the empty cast of the L3, on days 6 and 12. *O*. *volvulus* L3 molt *in vitro* usually by day 6 [28]. To control for possible changes in the time when molting is optimal in the presence of naive human neutrophils or monocytes, we observed the molting in control and experimental wells through day 12. Viability of the larvae was determined on day 12 by MTT (3-(4,5 dimethylthiazol-2yl)-2,5 diphenyl tetrazolium bromide) staining as previously described [13]. The experiments were done in triplicates and repeated twice on separate days. Results presented are the mean ± SD of two experiments.

To test whether contact between larvae, the antigen-specific monospecific antibodies and the monocytes was essential for their effect on molting, the inhibition of L3 molting assays in the presence of naïve monocytes were repeated using 96-well plates having trans-well trays with 0.4 μm pore size sterile polycarbonate membrane (Sigma). The L3s were distributed to designated wells under the trans-well trays of the 96-well plate per treatment group in complete media as mentioned previously, while the 2×10^5^ normal monocytes were added to each of the trans-well trays in 50 μL of complete media. The anti-*Ov*-103 or anti-*Ov*-RAL-2 purified monospecific antibodies (25 μL of 1:500,000 endpoint titer) or the anti-*Ov*-ASP-1 control antibodies were added (25 μL) under the trans-well trays where the L3s were added. Complete medium without antibodies was also included as a control. The 96-well plates were incubated at 37°C in a 5% CO_2_ incubator and the molting of L3 to L4 was monitored over the 12 days of culture. On day 12 the molting and survival was determined as described above. The experiment was done in triplicate wells and the results represent the mean ± SD of the three wells per condition.

### Statistical analysis

Data generated in the *in vivo* mouse model consisted of using 5–6 mice per group and the experiments were performed at least twice with consistent results between experiments. Data were analyzed using Systat v.11 (Systat Inc, Evanstown, IL) software using multifactorial analysis of variance ANOVA with Fishers Least Significant Different test for post hoc analysis. Antibody responses and cytokine/chemokine data were analyzed by Mann-Whitney U test. Correlation of antigen specific antibody responses in infected individuals with age was performed using Spearman correlation. The *in vitro* inhibition of L3 molting assay data with mono-specific antibodies was analyzed by One-way ANOVA with Tukey’s multiple comparisons test, while data with NH and INF pooled sera was analyzed by Mann-Whitney test respectively using GraphPad Prism software v.6 (San Diego, CA). Probability values less than 0.05 were considered statistically significant.

## Results

### *Ov*-103 and *Ov*-RAL-2 do not induce protective immunity in IgG deficient AID^-/-^ mice

**Fig 1:**
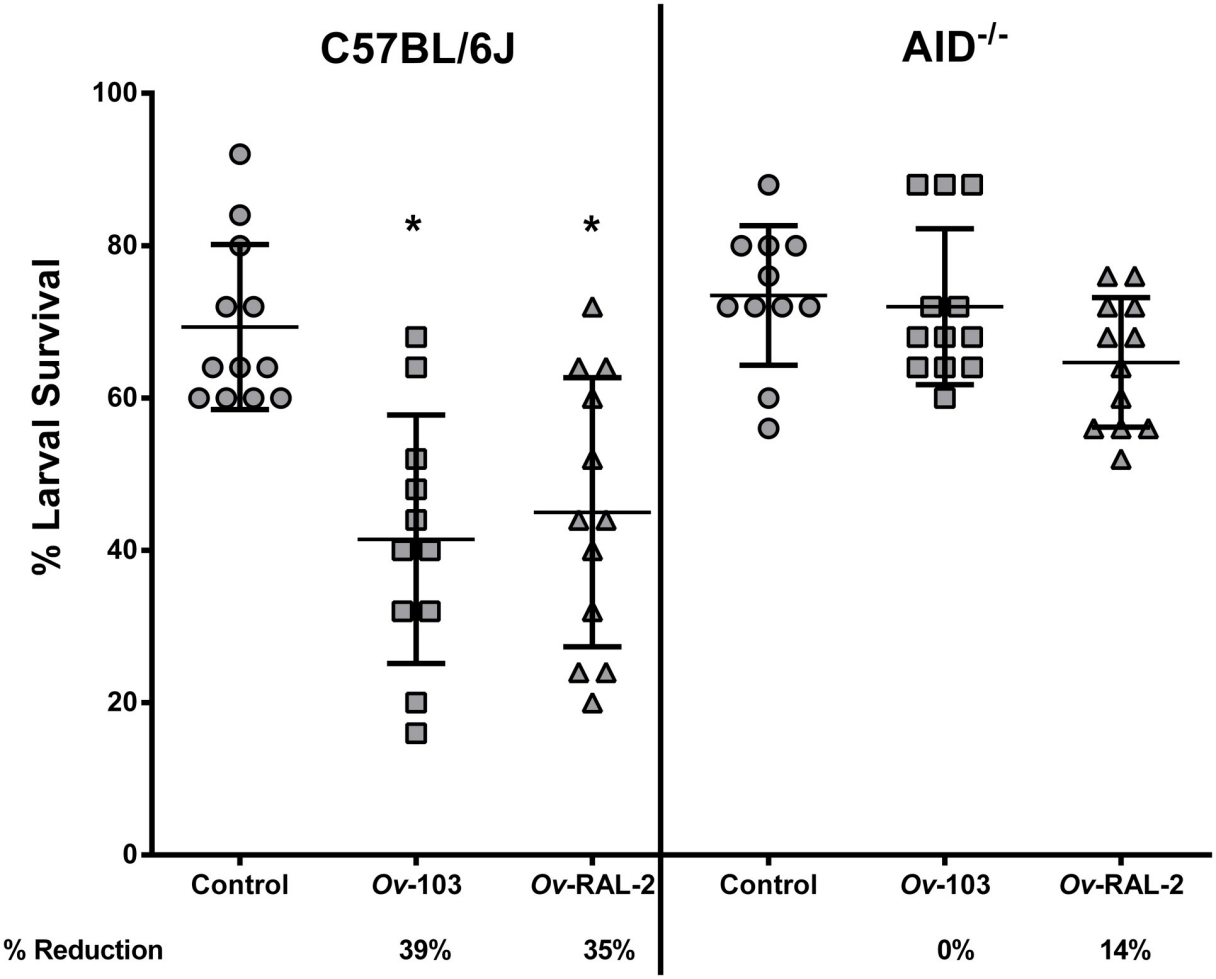
Immunization of C57BL/6J and AID^-/-^ mice with *Ov*-103 or *Ov*-RAL-2 vaccine antigens with alum as the adjuvant. The effect of immunization with *Ov*-103 or *Ov*-RAL-2 antigens adjuvanted with alum on the development of protective immunity to *O*. *volvulus* larvae was compared between C57BL/6J and AID^-/-^ mice. Each dot represents percent larval survival from an individual animal. Data lines in each group are mean ± standard deviation. Asterisk represents significant statistical difference in percent larval recoveries with *p* value ≤ 0.05. Protection is determined by the % Reduction = percent reduction in parasite survival in immunized mice as compared to controls. The data presented are from two independent experiments, n = 5–6 mice per group in each experiment.

C57BL/6J and AID^-/-^ mice were immunized with the recombinant antigens *Ov*-103 or *Ov*-RAL-2, with alum as the adjuvant. As expected statistically significant levels (*p* < 0.05) of protective immunity were seen in C57BL/6J mice immunized with *Ov*-103 or *Ov*-RAL-2; reduction of parasite survival ranged from 35%-39%. In contrast, *Ov*-103 immunized AID^-/-^ mice had no percent reduction of parasite survival, while *Ov*-RAL-2 immunized AID^-/-^ mice had only 14% reduction of parasite survival, suggesting that the mice strain which lacked the ability to produce IgG, did not develop statistically significant levels of protective immunity regardless of the vaccine composition (Fig 1).

Host cell recruitment to the parasite microenvironment was measured by differential cell analysis of the cells migrating into the diffusion chambers in which the worms were implanted. No differences were seen in the type or number of cells seen in diffusion chambers implanted in control and immunized mice. Nor was there a difference between cell migration into diffusion chambers implanted in C57BL/6J and AID^-/-^ mice. In diffusion chambers recovered from C57BL/6J mice, 9.2 ± 9.1 ×10^4^ cells were recovered in each diffusion chamber and in AID^-/-^ mice 10.6 ± 10.3 ×10^4^ cells were recovered. The cell composition in diffusion chambers in C57BL/6J mice was 34 ± 22% neutrophils, 2 ± 6% lymphocytes, 64 ± 23% monocytes and 1 ± 5% eosinophils, and in AID^-/-^ mice 28 ± 18% neutrophils, 1 ± 1% lymphocytes, 71 ± 18% monocytes and 1 ± 2% eosinophils.

Larvae recovered from control and immunized C57BL/6J and AID^-/-^ mice were measured and compared to infective L3. Worms recovered from both the control and immunized C57BL/6J and AID^-/-^ mice had grown over the 3 weeks implanted in mice to approximately the same size with a mean of length of 655 ± 38 μm as compared to infective L3 with a mean length of 533 ± 65 μm.

### Antigen-specific IgG1 is present in immunized C57BL/6J but not AID^-/-^ mice

Antigen-specific ELISA’s were performed to measure IgM and IgG1 levels in immunized C57BL/6J and AID^-/-^ mice. We tested only IgG1 as this was the dominant antibody subclass induced in the alum-adjuvanted vaccines [7]. Immunized C57BL/6J and AID^-/-^ mice developed antigen-specific IgM responses, with responses in immunized AID^-/-^ mice equivalent to those seen in C57BL/6J mice. IgG1 responses to *Ov*-103 and *Ov*-RAL-2 were elevated in C57BL/6J mice immunized with the antigens *Ov*-103 and *Ov*-RAL-2. As was expected, there was no measurable antigen specific IgG1 response to either antigen in immunized AID^-/-^ mice (Table 1).

**Table 1:** Antigen-specific IgM and IgG1 in immunized C57BL/6J and AID^-/-^ mice.

	C57BL/6J		AID-/-	
	*Ov*-103	*Ov*-RAL-2	*Ov*-103	*Ov*-RAL-2
**IgM**^a^	451 ± 645	11,057 ± 17,445	434 ± 136	1,214 ± 1,040
**IgG1**^a^	13,174 ± 8,847	190,240 ± 83,902	X	X

^a^Data represents endpoint titers in mean ± SD

### *Ov*-103 and *Ov*-RAL-2 induce Th2 cytokine responses in AID^-/-^ as in C57BL/6J mice

T cell responses in immunized mice were determined by measuring cytokine production by spleen cells recovered from immunized mice and re-stimulated *ex vivo* with each of the vaccine antigens. The dominant T cell responses in immunized C57BL/6J and AID^-/-^ mice was Th2 in nature, based on elevated IL-4 and IL-5 cytokine responses to both *Ov*-103 and *Ov*-RAL-2 (S1 Fig). Thus, the T cell responses elicited by both antigens in these mice were independent of the ability of the AID^-/-^ mice to produce antibodies.

### Antibody responses against *Ov*-103 and *Ov*-RAL-2 are elevated in the PI and in INF who developed concomitant immunity with age

**Fig 2:**
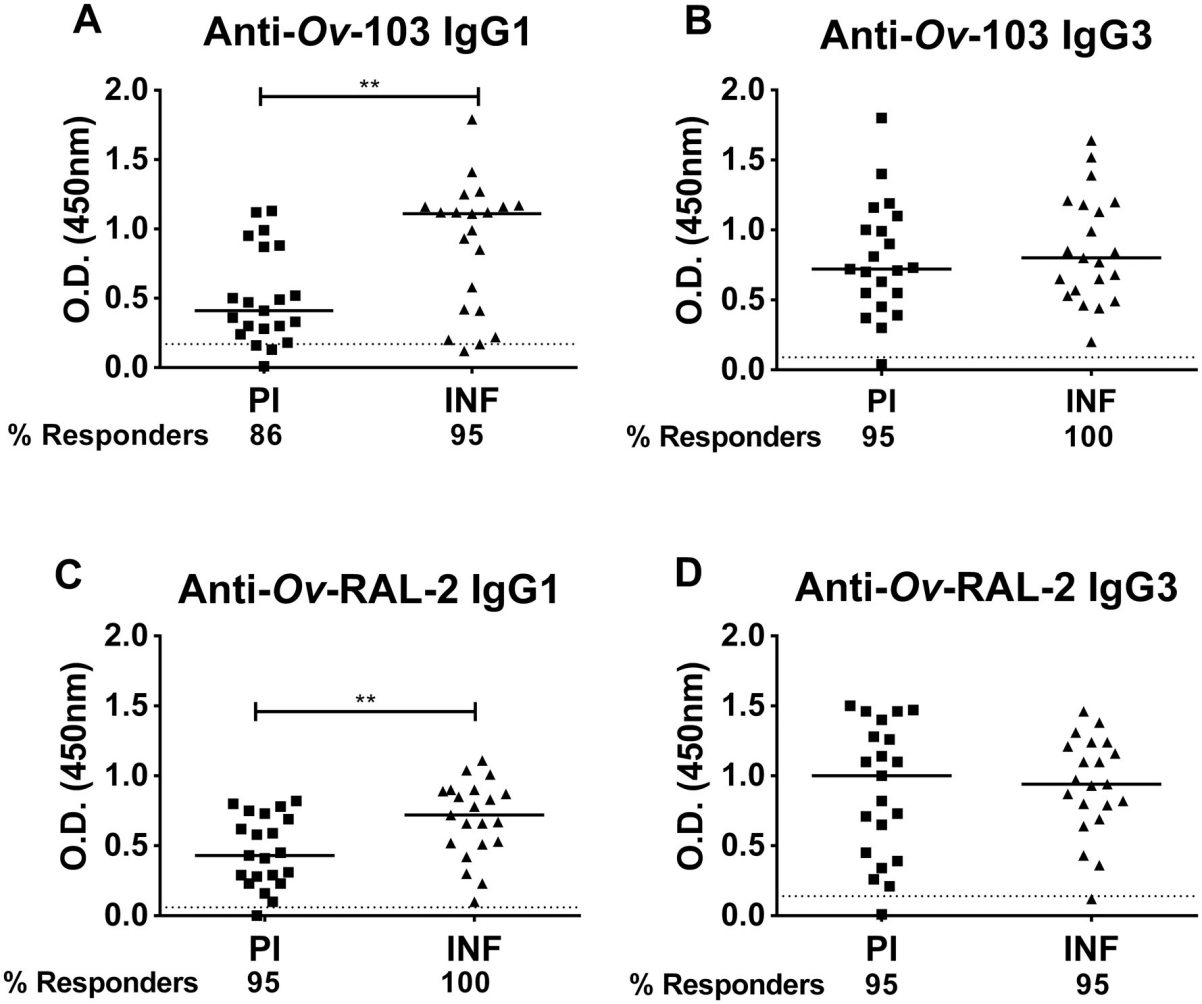
Both PI and INF individuals have high anti-*Ov*-103 and anti-*Ov*-RAL-2 specific IgG1 and IgG3 antibody responses. (A, B) anti-*Ov*-103 and (C, D) anti-*Ov*-RAL-2 antigen-specific IgG1 and IgG3 antibody responses were measured in PI (n = 21) and INF (n = 21) individuals at 1:100 dilution. The percentage of responders to *Ov*-103 and *Ov*-RAL-2 antigens was also significantly high in both PI and INF. Responders are individuals having IgG1 or IgG3 OD values higher than mean + 3*SD of normal human sera (indicated as dotted line). Analysis was done using the Mann-Whitney test (**, *p* < 0.01).

To test whether humoral immune responses against the two defined vaccine antigens are also relevant to protection in humans, we tested for *Ov*-103 and *Ov*-RAL-2 antigen-specific IgG1 and IgG3 responses (at 1:100 dilution) in sera from *O*. *volvulus*-exposed and protected individuals classified as putatively immune, and in infected individuals who developed concomitant immunity with age. These sera samples were previously analyzed and found to contain elevated cytophilic antibodies specific to L3 as well as to other recombinant vaccine antigens such as *Ov*-CPI-2, *Ov*-ALT-1, and *Ov*-ASP-1 [17, 24, 26]. First, the anti-*Ov*-103 and anti-*Ov*-RAL-2 specific IgG1 and IgG3 antibody responses were determined in PI and age matched INF individuals (Fig 2). Both PI and INF individuals have high levels of IgG3 cytophilic antibodies to both *Ov*-103 and *Ov*-RAL-2 antigens (Fig 2), while the IgG1 response was significantly higher in the INF to both proteins. Notably, the number of IgG1 and IgG3 responders to *Ov*-103 and to *Ov*-RAL-2 antigens was similarly high in both PI and INF groups (>86% IgG1 responders to both antigens; >95% IgG3 responders to both antigens) (Fig 2).

**Fig 3:**
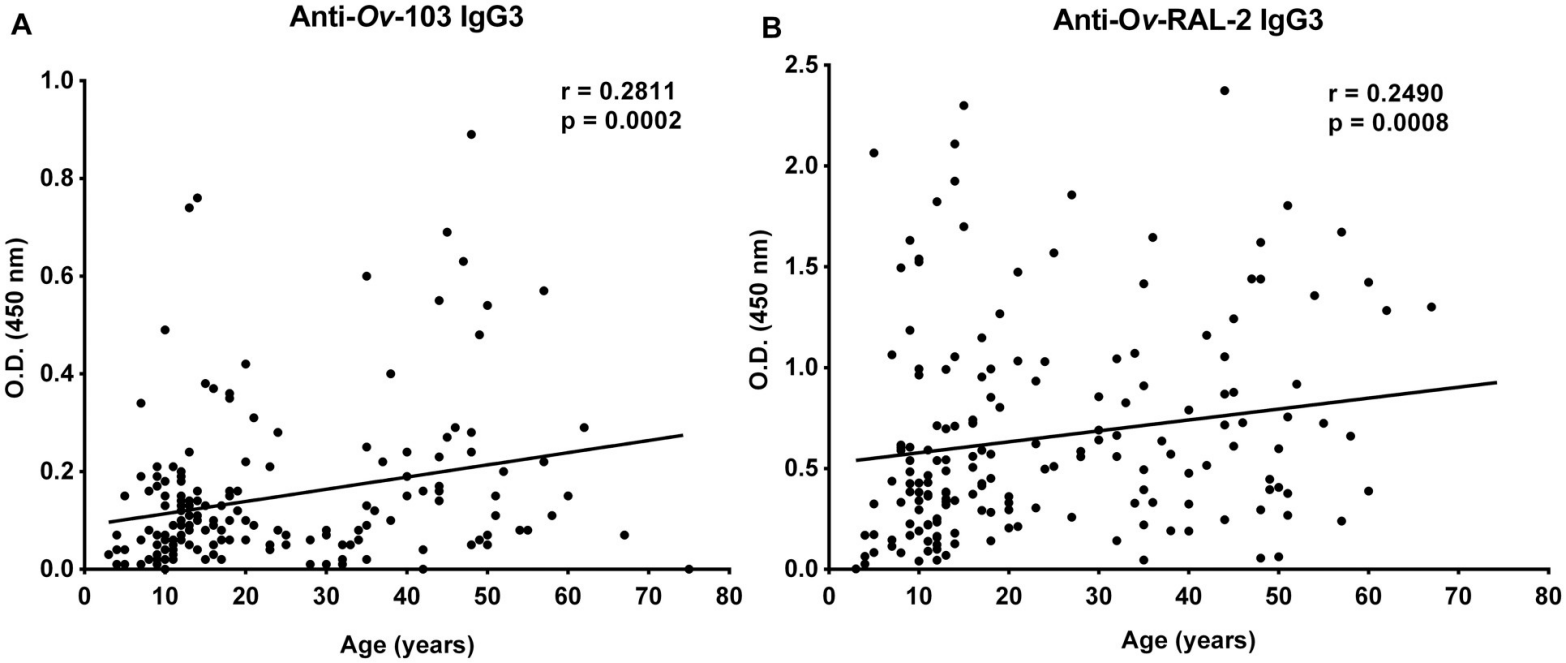
Correlation of anti-*Ov*-103 and anti-*Ov*-RAL-2 antigen-specific IgG3 antibody responses in infected individuals with age. The anti-*Ov*-103 (A) and anti-*Ov*-RAL-2 (B) antigen-specific IgG3 antibody responses in sera (1:100 dilution) from 167 infected individuals were analyzed using Spearman correlation.

In previous studies [17] we have shown that in INF that have developed concomitant immunity with age, the IgG3 responses against crude L3 proteins as well as against *Ov*-ALT-1 or *Ov*-CPI-2 were positively correlated with age, while the IgG1 responses were elevated regardless of age [17, 24]. Similar to the previous observations, the IgG1 responses against *Ov*-103 (r = 0.1285, *p* = 0.0970) and *Ov*-RAL-2 (r = -0.06223, *p* = 0.4066) were elevated regardless of age (S3 Fig) while the anti-*Ov*-103 and anti-*Ov*-RAL-2 specific IgG3 responses in the INF increased with age in response to *Ov*-103 (r = 0.2811, *p* = 0.0002) and to *Ov*-RAL-2 (r = 0.2490, *p* = 0.0008). As this association is not very strong, even though significant, we have reanalyzed these data and compared the antibody responses in the INF individuals above 15 years of age vs. below the age of 15. We have shown previously in the same cohort of individuals that the number of skin microfilariae plateaus in the INF by ages 10–15 years of exposure to *O*. *volvulus* [17], which suggested that these individuals might have developed a means of limiting acquired new infections while having a stable patent infection (microfilariae positive). The new analysis has shown that INF above 15 years of age had significantly higher IgG3 antibody responses to *Ov*-103 and *Ov*-RAL-2 compared to INF individuals below 15 years of age, further supporting the association of anti-*Ov*-103 and *Ov*-RAL-2 antigen-specific IgG3 antibody responses with age (Fig 3). In comparison, no significant differences were observed with the IgG1 antibody responses to the two antigens in INF below or above the age of 15 (S2 Fig).

These observations suggest that the elevated antibody responses to these two vaccine candidates (Fig 3) are also associated with the concomitant immunity that develops in infected individuals who are exposed to multiple *O*. *volvulus* L3 infections over the years.

### Association of chemokine responses with protection in the putatively immune and infected individuals with concomitant immunity

Chemokines that recruit and activate innate cells upon infection have been shown to play an important role in the immune responses in occult *O*. *volvulus* infections [29]. Based on our previous mouse studies [7], it appeared that chemokines associated with chemotaxis of neutrophils (KC, MIP-1α), monocyte/macrophages (MCP-1, MIP1β), and eosinophils (Eotaxin) are also part of the multifactorial immune responses associated with protection induced by *Ov*-103 and *Ov*-RAL-2 vaccines [7]. Therefore, to explore whether this might also be reflected in the PI and INF individuals with protective immune responses to larval proteins, we analyzed the plasma levels of 8 chemokines (Eotaxin, GM-CSF, KC, IP-10, MCP-1, MIP-1α, MIP-1β and RANTES) in both groups. Notably, only the chemokine levels of KC (neutrophils), MCP-1 and MIP-1β (monocyte/macrophages) and IP-10, an IFN-γ-inducible protein that is a chemoattractant for monocytes and activated T cells [30, 31], were significantly elevated in both INF and PI individuals (presented as % of responders), whereas no significant levels of the other chemokines were observed (Table 2).

**Table 2:** The percentage of responders in PI and INF individuals studied in chemokine responses.

	PI (n = 18)		INF (n = 17)	
Chemokines	# responders^a^	% responders^a^	# responders^a^	% responders^a^
**Eotaxin**	3	17	3	18
**GM-CSF**	4	22	0	0
**KC**	16	89	14	82
**IP-10**	16	89	16	94
**MCP-1**	17	94	16	94
**MIP-1α**	3	17	3	18
**MIP-1β**	13	72	11	65
**RANTES**	0	0	0	0

^a^Responders represent individuals having chemokine levels of more than mean + 3*SD of normal human sera.

### Differential ability of monospecific human anti-*Ov*-103 and anti-*Ov*-RAL-2 antibodies to inhibit the molting of *O*. *volvulus* L3 in the presence of naïve human neutrophils or monocytes

**Fig 4:**
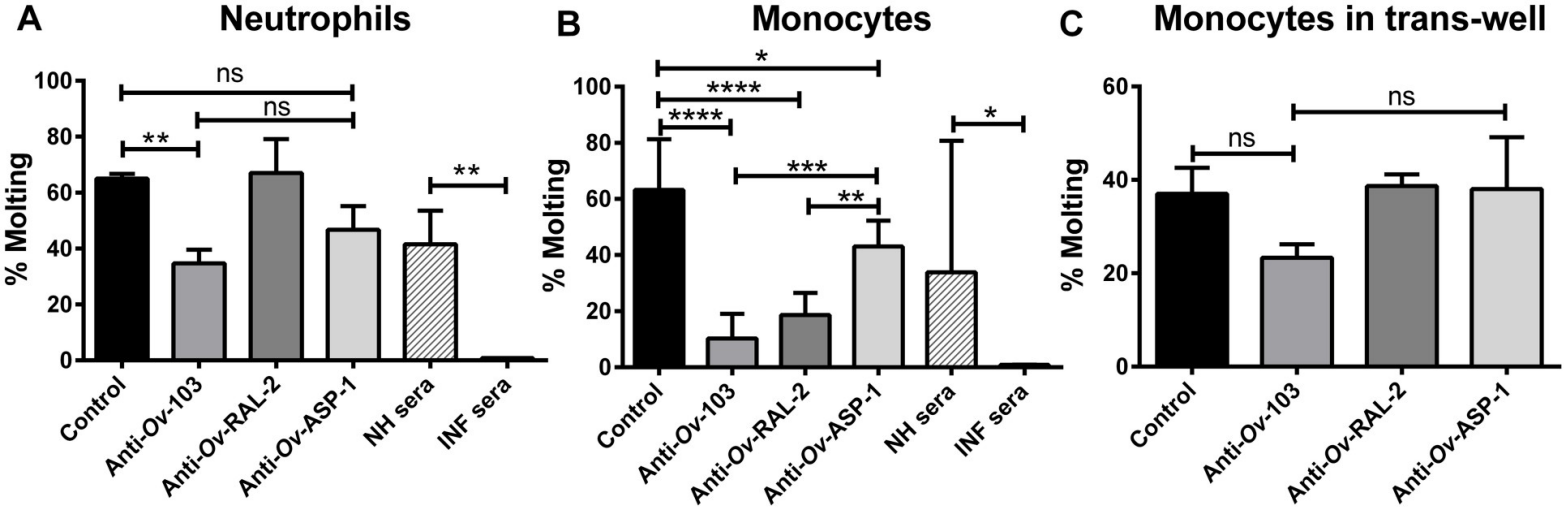
Effects of purified monospecific anti-*Ov*-103 or anti-*Ov*-RAL-2 antibodies on the molting of *O*. *volvulus* L3 larvae in the presence of naïve human neutrophils (A), monocytes (B), or with monocytes in trans-well (C). Control: Larvae + neutrophils or Larvae + monocytes; anti-*Ov*-ASP-1 monospecific antibodies were used as the non-related *Ov*-103 or *Ov*-RAL-2 antibody control. Pooled sera from NH and INF individuals were used for comparison. Analysis was done using one-way ANOVA with Tukey’s multiple comparisons test (with monospecific antibodies) and Mann-Whitney test (NH vs INF sera): *, *p* < 0.05; **, *p* < 0.01; ***, *p* < 0.001, and ****, *p* < 0.0001, ns–non-significant.

To test whether the elevated anti-*Ov*-103 and anti-*Ov*-RAL-2 cytophilic antibodies present in the PI and the infected individuals can function in ADCC, *O. volvulus* L3 were cultured in the presence of human naïve neutrophils or monocytes and monospecific human anti-*Ov*-103 or anti-*Ov*-RAL-2 antibodies. Monospecific anti-*Ov*-ASP-1 antibodies were used as negative control (these antibodies did not cross-react with *Ov*-103 or *Ov*-RAL-2). Molting levels were observed on day 6 and on day 12 (S1 Table). The data in Fig 4 represent the L3 molting observed on day 12. Anti-*Ov*-103 antibodies in the presence of neutrophils reduced significantly the molting by 46% in comparison to the control wells (65% of molting), while anti-*Ov*-RAL-2 antibodies did not inhibit the molting (Fig 4A). The inhibition of molting (calculated as % molting in control wells—% molting in test wells / % of molting in control wells) in the presence of anti-*Ov*-103 was only 25.5% lower than that in the presence of anti-*Ov*-ASP-1 and not significant, which is potentially due to the ability of the anti-*Ov*-ASP-1 antibodies to also partially inhibit the molting of L3 in the presence of neutrophils (27.7%) when compared to control wells (Fig 4A, S1 Table). Pooled sera from three INF individuals completely inhibited molting in the presence of neutrophils, as compared to the 42% molting observed for worms cultured in pooled sera from normal healthy individuals (NH) (Fig 4A, S1 Table). Both anti-*Ov*-103 and anti-*Ov*-RAL-2 antibodies, in the presence of naïve human monocytes, however, significantly inhibited the molting of larvae; 84% and 69%, respectively, when compared with control (Fig 4B, S1 Table). Anti-*Ov*-ASP-1 antibodies also had the ability to significantly inhibit molting in the presence of monocytes (31.7%) when compared to control wells. Nevertheless, when compared to anti-*Ov*-ASP-1 antibodies, both anti-*Ov*-103 and anti-*Ov*-RAL-2 antibodies in the presence of naïve human monocytes still significantly inhibited molting of larvae by 76.7% and 55.8%, respectively (Fig 4B, S1 Table). Pooled sera from three INF individuals also inhibited molting completely in the presence of monocytes, as compared to 67% molting observed in pooled sera from NH individuals (Fig 4B, S1 Table). None of the monospecific antibodies and cell combinations tested in this study affected significantly the survival of larvae on day 12 as compared to control wells.

To test whether the inhibition of molting by the anti-*Ov*-103 and anti-*Ov*-RAL-2 antibodies in the presence of the naïve human monocytes required contact with the larvae, assays were performed using trans-wells, with the larvae placed in the bottom well and the monocytes at the top well. Anti-*Ov*-103 antibody-mediated inhibition of molting was found to be partially dependent on contact, with 37% inhibition of molting observed when the monocytes were separated from the larvae as compared to controls (Fig 4C). When the cells were in contact with the larvae the inhibition in the presence of anti-*Ov*-103 was 84% on day 12 (Fig 4B, S1 Table). In contrast, anti-*Ov*-RAL-2 antibodies did not mediate any inhibition of molting when the monocytes were not in contact with the larvae (Fig 4C).

### Protective immunity in mice induced by *Ov*-103 and *Ov*-RAL-2 requires cell contact

**Fig 5:**
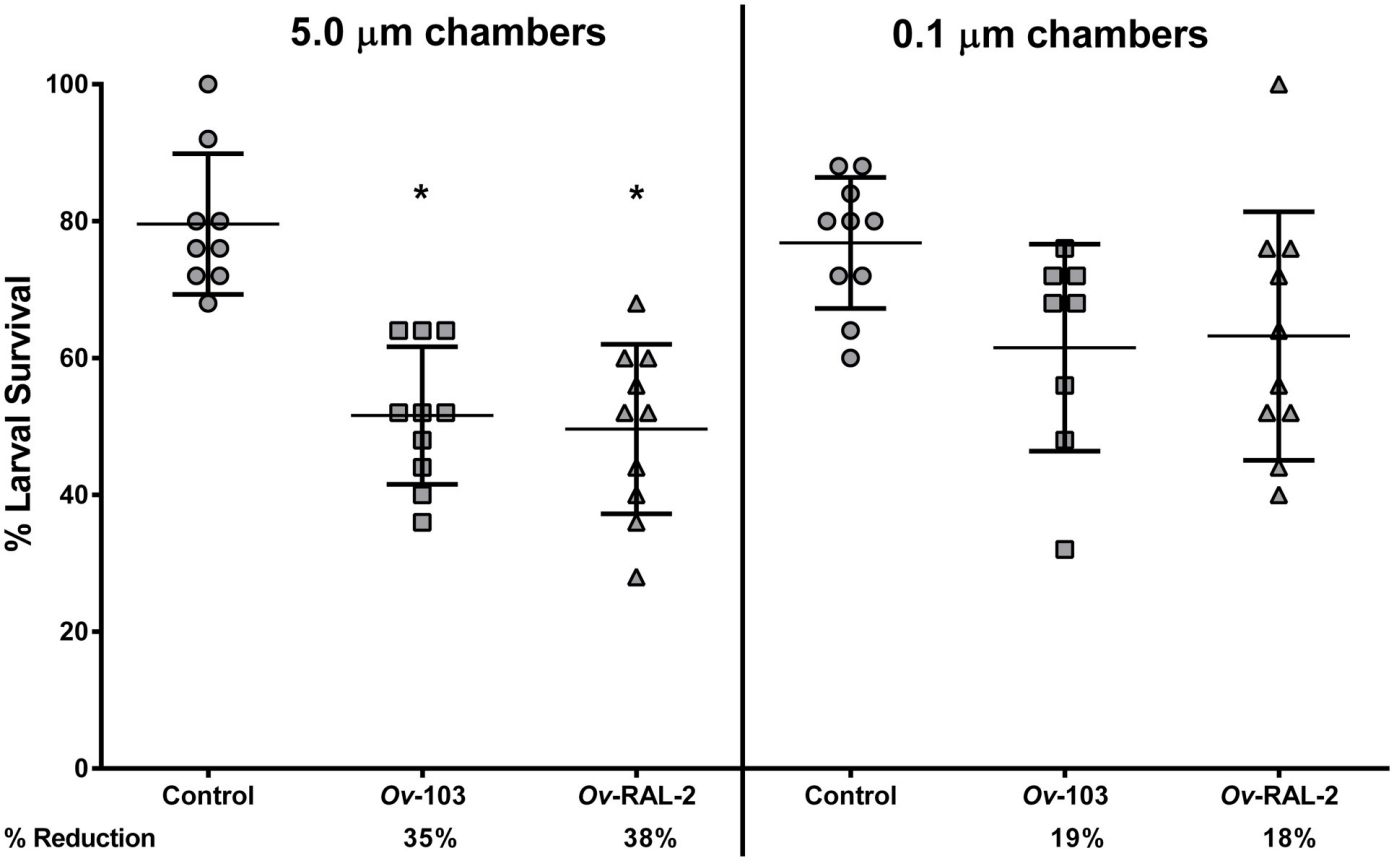
Percentage of larval survival implanted within 5.0 μm and 0.1 μm diffusion chambers in C57BL/6J mice immunized with alum-adjuvanted *Ov*-103 or *Ov*-RAL-2 vaccines. The effect of immunization of C57BL/6J mice with *Ov*-103 or *Ov*-RAL-2 antigens adjuvanted with alum on the development of protective immunity to *O*. *volvulus* larvae implanted within diffusion chambers, covered with 5.0 μm or 0.1 μm membranes, was measured 3 weeks post challenge. Each dot represents percent larval recovery from an individual animal. Data lines in each group are mean ± standard deviation. Asterisk represents significant statistical difference in larval recoveries with *p* value ≤ 0.05. Protection is determined by the % Reduction = percent reduction in parasite survival in immunized mice as compared to controls. The data presented are from two independent experiments, n = 5–6 mice per group in each experiment.

C57BL/6J mice were immunized with either *Ov*-103 or *Ov*-RAL-2 and then received implanted challenge infections in diffusion chambers. The diffusion chambers were constructed with membranes of either 5.0 μM pore-sizes that allowed cells to enter the parasite’s microenvironment or with membranes with 0.1 μM pore-sizes that completely blocked cell entry. It was observed that a statistically significant level of reduction in larval survival within the diffusion chambers only occurred when cells could enter the parasite’s microenvironment; blocking cell entry into the diffusion chamber blocked the protective effects of the immune responses induced by each of the alum-adjuvanted vaccine antigens (Fig 5).

## Discussion

Parallel studies in mice and humans support the conclusion that protective immunity induced by the antigens *Ov*-103 and *Ov*-RAL-2 is dependent on antibodies. Based on the mouse studies, cell contact is also required for the effect on larval survival. In comparison, in the human studies cell contact (monocytes) is required for inhibition of molting by some antibody cell interactions (anti-*Ov*-RAL-2) but only partially by the other (anti-*Ov*-103).

AID^-/-^ mice, which are unable to undergo class-switch recombination, did not develop protective immunity following immunization with alum-adjuvanted *Ov*-103 or *Ov*-RAL-2 vaccines. These mice did develop an antigen-specific IgM response but not an IgG1 response. It was shown [32] that the B cells develop normally but when there is an antigen stimulation, a much stronger IgM response develops in the AID^-/-^ as compared to WT mice; regardless, the B cells cannot complete class switching. Apparently, IgM antibodies against *Ov*-103 or *Ov*-RAL-2 were not sufficient to kill *O*. *volvulus* larvae as compared to other nematode parasites which are also susceptible to killing through IgM dependent mechanisms [33–36]. The critical role of IgG in killing L3 within the diffusion chambers was reinforced by the observation that T cell responses in immunized AID^-/-^ mice shared the Th2 cytokine bias seen in wild-type mice. Moreover, the number and types of cells recruited to the parasite microenvironment were equivalent to those observed in the vaccinated C57BL/6J mice. The only obvious immune defect in the immunized AID^-/-^ mice was their IgG1 antibody response. It was therefore concluded that protection induced by vaccination of mice with alum-adjuvanted *Ov*-103 or *Ov*-RAL-2 is IgG1 antibody-dependent. Larvae recovered from control and immunized C57BL/6J and AID^-/-^ mice were measured and had equal growth rates, indicating that there was larval killing three weeks post challenge, but no developmental regulation, in the immunized mice. Finally, studies in immunized C57BL/6J mice demonstrated that the presence of antibody alone was not sufficient to affect the survival of worms *in vivo*; cell contact as part of a presumptive ADCC reaction was a critical component of the protective mechanism against challenge infection.

Analysis of human sera from PI and INF individuals showed that both groups had similar percentage of individuals with anti-*Ov*-103 and *Ov*-RAL-2 antigen-specific IgG1 and IgG3 responses, albeit a higher IgG1 reactivity was observed in the infected individuals. The anti-*Ov*-103 and anti-*Ov*-RAL-2 IgG3 responses in the INF individuals increased significantly with age, which is consistent with the development of concomitant immunity in these individuals over time [24]. The presence of cytophilic IgG3 antibody responses against *O*. *volvulus* larval antigens has been shown to be associated with immune protection in onchocerciasis [17, 24, 37, 38]. It was postulated that one of the mechanisms through which cytophilic IgG or IgE antibodies participate in the protective immune responses in humans is by inhibiting early development of L3 to L4, and/or by killing these parasites through ADCC [16]. Neutrophils, in the presence of sera from PI and INF were shown to be able to inhibit molting of *O*. *volvulus* larvae [13] and kill mf *in vitro* [39], and neutrophils in the presence of monospecific anti-*Ov*-CPI-2 antibodies, inhibited molting and also killed the L3 [24].

One of the essential aspects of the ADCC mechanism is the recruitment of effector cells bearing Fc receptors, such as monocytes/macrophages, neutrophils and eosinophils that recognize and target IgG, IgA or IgE antibody coated parasitic worms by releasing their lysosomal or granular proteins [10, 40–42]. Antibodies and ADCC were also found to be an important part of protective effector mechanisms in the *O*. *volvulus* attenuated L3 mouse model and were predicted to also function in humans living in highly endemic regions of onchocerciasis [13, 16, 39, 43]. In the present study, we found the majority of both PI and INF had elevated plasma levels of the chemokines KC, IP-10, MCP-1 and MIP-1β. KC is associated with the chemotaxis of neutrophils, while IP-10 is associated with the chemotaxis of monocytes/macrophages, dendritic cells, T cells and natural killer cells. MCP-1 and MIP-1 β are associated with the chemotaxis of monocytes [30, 31, 44–48]. Interestingly, in occult mf negative *O*. *volvulus* infections, serum levels of pro-inflammatory chemokines MCP-1, MIP-1α, MIP-1β, MPIF-1 and CXCL8 were also enhanced in comparison to sera from infection-free controls [29]. Notably, in previous vaccination studies it was found that alum-adjuvanted *Ov*-103 vaccinated and protected C57BL/6J mice had elevated chemokines associated with activation and chemotaxis of neutrophils (KC) and eosinophils (Eotaxin) within the diffusion chambers. In comparison, MCP-1, MIP-1β (associated with chemotaxis of monocytes) and MIP-1α (associated with chemotaxis of granulocytes, particularly neutrophils) were elevated within diffusion chambers recovered from protected mice immunized with the alum-adjuvanted *Ov*-RAL-2 vaccine. Because of these differences, it was proposed that the mechanism of protective immunity induced by *Ov*-RAL-2 might differ from that induced by *Ov*-103 [7].

Previous studies have shown that neutrophils purified from naïve PBMCs were able to inhibit molting and kill the L3 in the presence of sera from both the PI and the INF [13]. To test whether anti-*Ov*-103 and anti-*Ov*-RAL-2 monospecific human antibodies are functional in ADCC, we tested them first with naïve human neutrophils. Interestingly, only the anti-*Ov*-103 antibodies significantly inhibited the molting of *O*. *volvulus* L3 to L4 larvae (46%), while anti-*Ov*-RAL-2 antibody had no effect. However, both anti-*Ov*-103 and anti-*Ov*-RAL-2 antibodies significantly inhibited the molting of L3 in the presence of the naïve monocytes. This is the first study, to the best of our knowledge, in which purified monocytes were used *in vitro* in ADCC assays with *O*. *volvulus* L3s. None of these antibodies, however, affected the survival of the larvae under the experimental conditions tested. Moreover, the anti-*Ov*-103 antibody-mediated inhibition of molting was found to be partially dependent on contact with the monocytes, suggesting that soluble cytotoxic factors secreted by the monocytes may also be involved in the partial inhibition of molting. The anti-*Ov*-RAL-2 antibodies could not block molting when monocytes were not in direct contact with the larvae, suggesting that inhibition of molting with the anti-*Ov*-RAL-2 antibodies is contact-dependent. Thus, as in the mouse studies, the effect of the human *Ov*-103 and *Ov*-RAL-2 antigen-specific antibody responses might function through distinct ADCC mechanisms, even though both antigens are similarly expressed on the surface and in the glandular esophagus of L3 [8].

The soluble factors released from monocytes that cooperate with the anti-*Ov*-103 antibodies to partially inhibit L3 molting *in vitro* were not identified in this study. Soluble factors that collaborate in the nematode and trematode killing process have been identified in other systems. *Strongyloides stercoralis* larvae trigger the release of extracellular DNA traps by human neutrophils and macrophages, which were essential for larval killing by these cells *in vitro* [49]. Eosinophils release major basic protein and polymorphonuclear neutrophils (PMN) release myeloperoxidase that also participate in killing *S*. *stercoralis* larvae [50]. PMN cells were also shown to expel large amounts of extracellular traps that bind and form aggregates around *B*. *malayi* mf *in vitro* [51]. Monocytes/macrophages from rat peritoneal lavage were able to mediate ADCC against newly excysted juvenile *Fasciola hepatica in vitro* in a nitric oxide dependent manner [42]. Future studies will attempt to identify the soluble factors that are be involved in the inhibition of *O*. *volvulus* molting *in vitro* by human effector cells.

Vaccine studies using the *B*. *malayi* infection model in gerbils have demonstrated that immunization with the *B*. *malayi* homologues of *Ov*-RAL2 or *Ov*-103 vaccine antigens induced protective immunity that was, at least in part, associated with the presence of antigen-specific antibodies that could kill *B*. *malayi* L3 *in vitro* in the presence of naïve peritoneal exudate cells [8]. The present study showed that antibodies are essential for the reduced survival of *O*. *volvulus* larvae three weeks post-challenge in mice immunized with *Ov*-RAL2 or *Ov*-103. Neutrophils in the presence of sera from PI and INF were shown to be effective at inhibiting *O*. *volvulus* larvae molting and killing L3 and mf *in vitro* [13, 14], and in the presence of monospecific anti-*Ov*-CPI-2 antibodies, inhibited development and killed the L3 [24]. Human monospecific antibodies against *Ov*-103 also killed mf *in vitro* in the presence of neutrophils [15]. It was thus surprising that human monospecific antibodies to *Ov*-RAL2 or *Ov*-103, in the presence of neutrophils or monocytes, only blocked molting but did not kill the worms (at least by day 12). A consistent observation in animal models of filarial infections is that following immunization, there are two manifestations of protective immunity. The first is inhibition of parasite development and the second is larval killing. This has been reported for *Litomosoides sigmodontis* [52], *Acanthocheilonema viteae* [53, 54], *B*. *malayi* [55] and *Dirofilaria immitis* [56, 57]. There are clearly differences in the immunologic characteristics between L3 and L4 of *O*. *volvulus* [28]. It has been postulated that growth retardation is a mechanism used by the immune response to keep the parasites in a stage that is more susceptible to the immune response. Therefore, from our *in vitro* experiments we speculate that the inhibition of molting of L3 by human monospecific antibodies and cells *in vitro* might be an early component of the protective immune response. Alternatively, killing of *O*. *volvulus* L3 by human monospecific antibodies to *Ov*-RAL-2 or *Ov*-103 may require more than one cell type to be present as observed with other worms [58] This might explain why there is killing of *O*. *volvulus* larvae *in vivo* in mice, and *B*. *malayi* L3 in *vitro* in the presence of naïve peritoneal exudate cells [8] yet no killing of *O*. *volvulus* L3 by human *Ov*-RAL2 or *Ov*-103 monospecific antibodies in the presence of either neutrophils or monocytes.

In conclusion, this study demonstrates that the antibody responses to *Ov*-103 and *Ov*-RAL-2 antigens are integral to the development of protective immunity to *O*. *volvulus* infections in both mouse model and in humans. We have also established that this protective immunity is multi-factorial, involving a collaborative role of antibodies, chemokines and innate cells. Moreover, *in vitro* ADCC assays have shown that anti-*Ov*-103 antibodies mediated inhibition of molting of L3 with both neutrophils and monocytes, and that it was partially dependent on monocyte contact unlike with anti-*Ov*-RAL-2 antibodies that inhibited molting only in the presence of monocytes and this inhibition was contact-dependent. These outcomes suggest that different mechanisms of protective immunity might be induced by the two vaccine candidate antigens. Although, we did not observe larvae killing with antibodies to the individual antigens by day 12, the observations *in vitro* may not represent the comprehensive range of effector mechanisms responsible for the reduction of *O*. *volvulus* larvae survival in mice and human. Regardless, it allowed us to identify some of the components associated with the immune responses against *Ov*-103 and *Ov*-RAL-2 antigens that are present in humans who develop protective immunity against *O*. *volvulus*. Future experiments in the mouse model and *in vitro*, including the identification of soluble factors elicited by monocytes co-cultured with mono-specific antibodies and L3, will support a better understanding of the mechanism of protective immunity that can be induced by the alum-adjuvated *Ov*-103 and *Ov*-RAL-2 vaccines. Knowing the vaccine-induced mechanism(s) of protective immunity will ultimately help verify that the appropriate functional immune responses are stimulated when such vaccines are used in human clinical trials.

## Supporting information

S1 Fig Cytokine production by spleen cells from immunized C57BL/6J and AID^-/-^ mice following re-stimulation *in vitro*. Spleen cells recovered from C57BL/6J and AID^-/-^ mice immunized with alum-adjuvanted *Ov*-103 or *Ov*-RAL-2 vaccine were re-stimulated with either *Ov*-103 or *Ov*-RAL-2 antigens *ex-vivo*. Cytokine levels in the culture supernatants were measured using Luminex assays. Values presented are fold increases in cytokine levels measured from cultures of spleen cells from immunized mice as compares to stimulated cells from control mice. n = 5–6 mice per group. Data presented are average from two independent experiments.
(TIF)

S2 Fig INF individuals older than 15 years of age have significantly higher anti-*Ov*-103 and anti-*Ov*-RAL-2-specific IgG3 antibody responses. (A) anti-*Ov*-103 and (B) anti-*Ov*-RAL-2 antigen-specific IgG1 and IgG3 antibody responses were measured in INF individuals ≤15 and ≥16 years of at 1:100 dilution. The dotted line indicates OD values of the mean + 3*SD of normal human sera. Analysis was done using the Mann-Whitney test (*, *p*<0.05, **, *p* < 0.01).
(TIF)

S3 Fig Correlation of anti-*Ov*-103 and anti-*Ov*-RAL-2 antigen-specific IgG1 antibody responses in infected individuals with age. The anti-*Ov*-103 (A) and anti-*Ov*-RAL-2 (B) antigen-specific IgG1 antibody responses in sera (1:100 dilution) from 167 infected individuals were analyzed using Spearman correlation.
(TIF)

S1 Table Percentage of molting *in vitro* with monospecific anti-*Ov*-103 or anti-*Ov*-RAL-2 antibodies in the presence of neutrophils and monocytes on day 6 and day 12. Control: Larvae + neutrophils or Larvae + monocytes; anti-*Ov*-ASP-1 was used as the *Ov*-103 or *Ov*-RAL-2 non-related antibody control. Pooled sera from normal healthy (NH) individuals or *O*. *volvulus* infected (INF) individuals. The experiments were done in triplicates and repeated twice on separate days. Results presented are the mean ± SD.
(DOCX)
