# Supplementary figures and images for: Antibody responses against the vaccine antigens Ov-103 and Ov-RAL-2 are associated with protective immunity to Onchocerca volvulus infection in both mice and humans
Source: PLoS Negl Trop Dis. 2019 Sep 16;13(9):e0007730. doi: 10.1371/journal.pntd.0007730 (PMC6762197; doi:10.1371/journal.pntd.0007730)

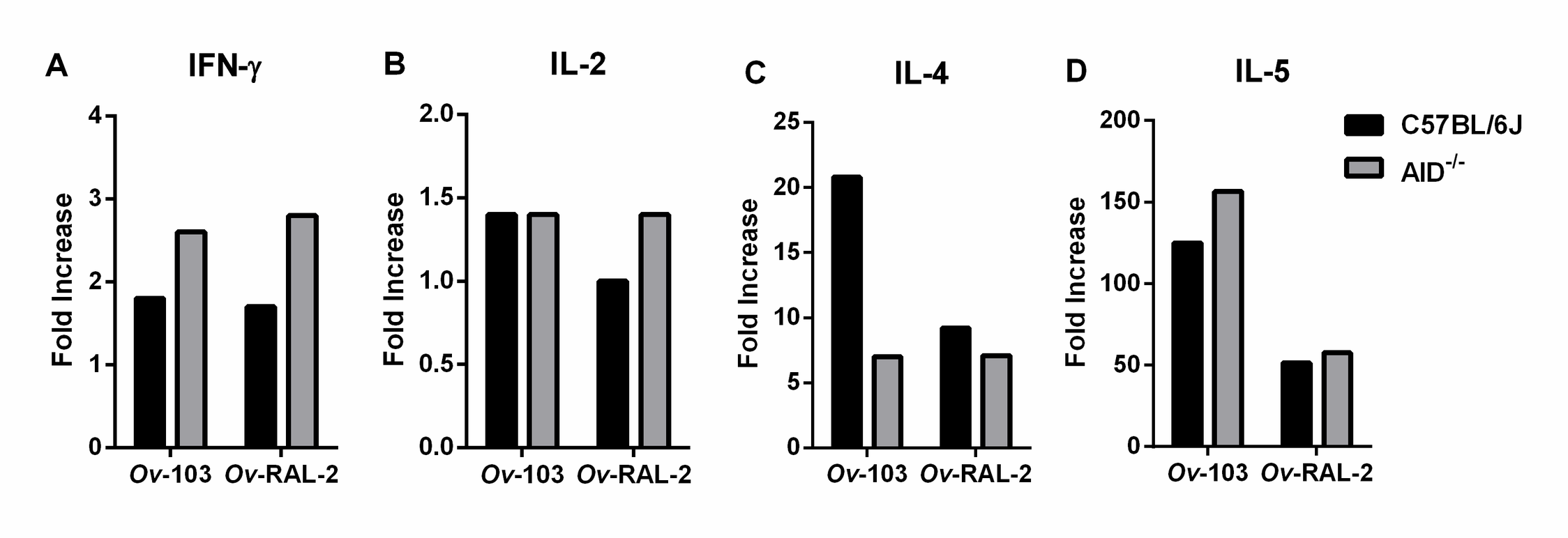

Supplement: S1 Fig — Spleen cells recovered from C57BL/6J and AID-/- mice immunized with alum-adjuvanted Ov-103 or Ov-RAL-2 vaccine were re-stimulated with either Ov-103 or Ov-RAL-2 antigens ex-vivo. Cytokine levels in the culture supernatants were measured using Luminex assays. Values presented are fold increases in cytokine levels measured from cultures of spleen cells from immunized mice as compares to stimulated cells from control mice. n = 5–6 mice per group. Data presented are average from two independent experiments. (TIF) [file pntd.0007730.s001.tif]

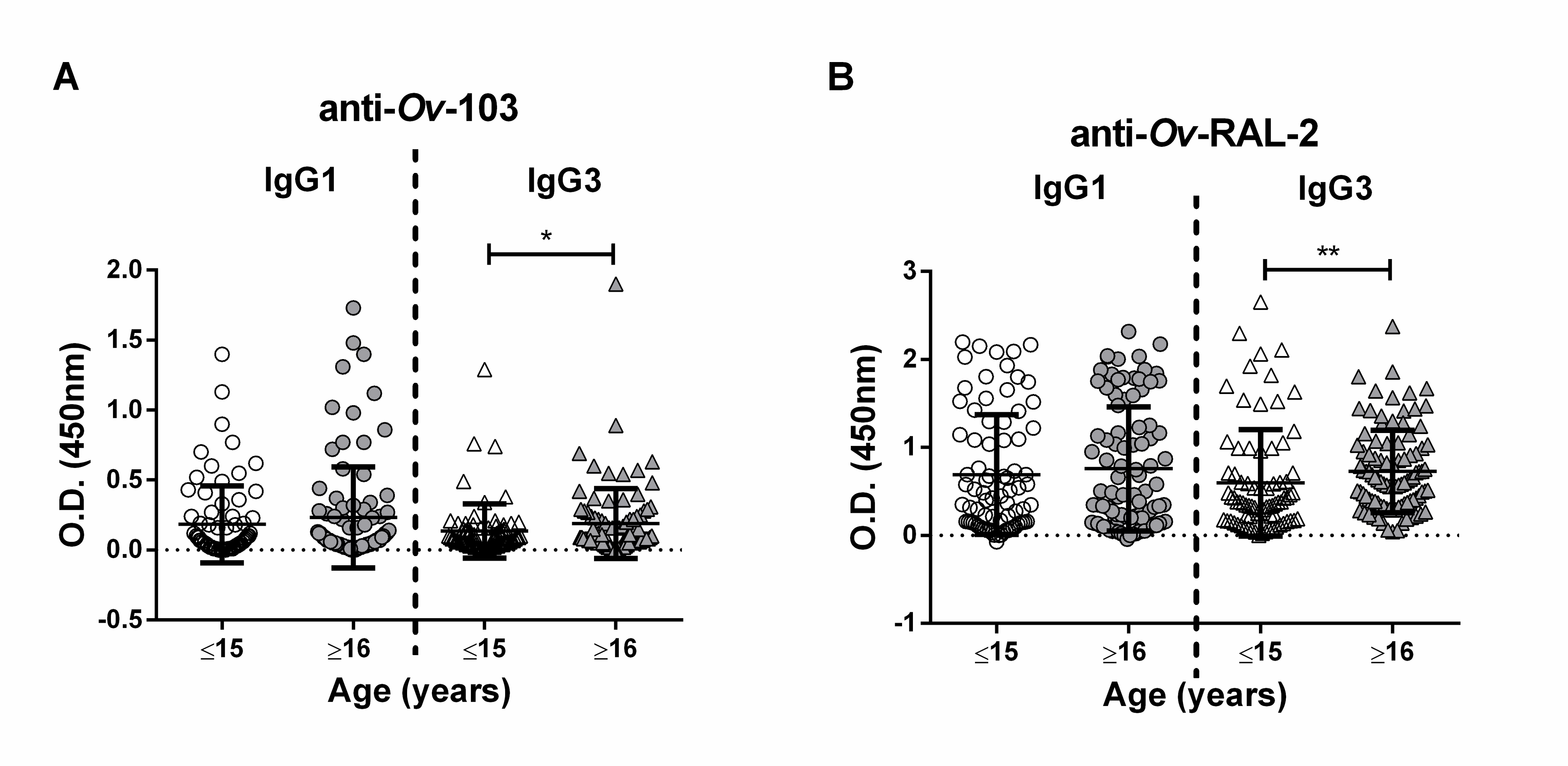

Supplement: S2 Fig — (A) anti-Ov-103 and (B) anti-Ov-RAL-2 antigen-specific IgG1 and IgG3 antibody responses were measured in INF individuals ≤15 and ≥16 years of at 1:100 dilution. The dotted line indicates OD values of the mean + 3*SD of normal human sera. Analysis was done using the Mann-Whitney test (*, p<0.05, **, p < 0.01). (TIF) [file pntd.0007730.s002.tif]

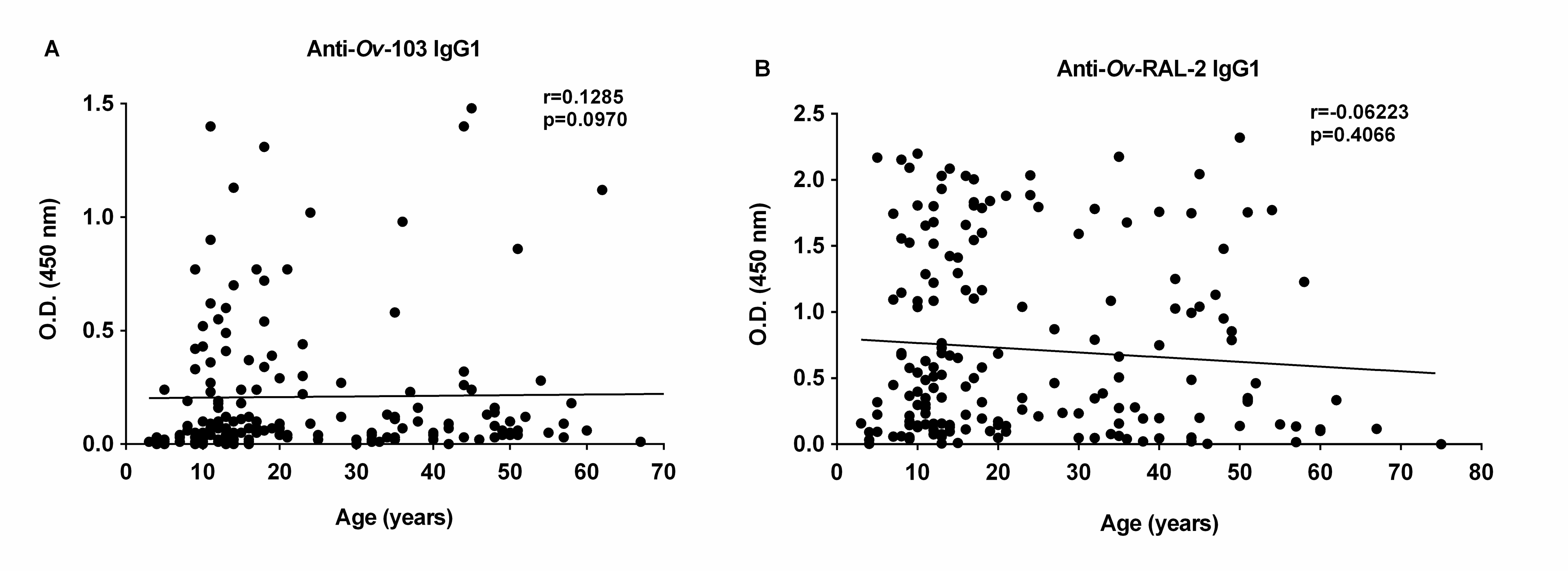

Supplement: S3 Fig — The anti-Ov-103 (A) and anti-Ov-RAL-2 (B) antigen-specific IgG1 antibody responses in sera (1:100 dilution) from 167 infected individuals were analyzed using Spearman correlation. (TIF) [file pntd.0007730.s003.tif]
