# Supplementary material for: Antibody responses against the vaccine antigens Ov-103 and Ov-RAL-2 are associated with protective immunity to Onchocerca volvulus infection in both mice and humans
Source: PLoS Negl Trop Dis. 2019 Sep 16;13(9):e0007730. doi: 10.1371/journal.pntd.0007730 (PMC6762197; doi:10.1371/journal.pntd.0007730)
Supplement: S1 Table — Control: Larvae + neutrophils or Larvae + monocytes; anti-Ov-ASP-1 was used as the Ov-103 or Ov-RAL-2 non-related antibody control. Pooled sera from normal healthy (NH) individuals or O. volvulus infected (INF) individuals. The experiments were done in triplicates and repeated twice on separate days. Results presented are the mean ± SD. (DOCX) [file pntd.0007730.s004.docx]

**S1 Table: Percentage of molting *in vitro* with monospecific anti-*Ov*-103 or anti-*Ov-*RAL-2 antibodies in the presence of neutrophils or monocytes on day 6 and day 12.**

|  | **% molting in the presence of neutrophils**^b^ | | **% molting in the presence of monocytes**^b^ | |
| --- | --- | --- | --- | --- |
|  | **Day 6** | **Day 12** | **Day 6** | **Day 12** |
| **Control**^a^ | 65 ± 2 | 65 ± 2 | 54 ± 16 | 63 ± 18 |
| **anti-*Ov*-103** | 35 ± 5 | 35 ± 5 | 5 ± 6 | 10 ± 9 |
| **anti-*Ov*-RAL-2** | 67 ± 12 | 67 ± 12 | 17 ± 7 | 19 ± 8 |
| **anti-*Ov*-ASP-1**^a^ | 47 ± 9 | 47 ± 9 | 40 ± 11 | 43 ± 9 |
| **NH sera**^c^ | 25 ± 12 | 42 ± 12 | 33 ± 47 | 33 ± 47 |
| **INF sera**^c^ | 0 | 0 | 0 | 0 |

^a^Control: Larvae + neutrophils or Larvae + monocytes; anti-*Ov*-ASP-1 was used as the non-related antibody control.

^b^Results presented are the mean ± SD.

^c^Pooled sera from normal healthy (NH) individuals or *O. volvulus* infected (INF) individuals.
